# Circulating insulin-like growth factor-1 and risk of lung diseases: A Mendelian randomization analysis

**DOI:** 10.3389/fendo.2023.1126397

**Published:** 2023-03-03

**Authors:** Sujing Jiang, Zhiyong Xu, Yueli Shi, Sibei Liang, Xinyuan Jiang, Mingshu Xiao, Kai Wang, Liren Ding

**Affiliations:** ^1^ Department of Respiratory and Critical Care Medicine, The Fourth Affiliated Hospital of Zhejiang University School of Medicine, Yiwu, China; ^2^ Department of Respiratory and Critical Care Medicine, The Second Affiliated Hospital of Zhejiang University School of Medicine, Hangzhou, China

**Keywords:** insulin-like growth factor-1, asthma, chronic obstructive pulmonary disease, lung cancer, idiopathic pulmonary fibrosis, Mendelian randomization

## Abstract

**Background:**

Insulin-like growth factor-1 (IGF-1) display a vital role in in the pathogenesis of lung diseases, however, the relationship between circulating IGF-1 and lung disease remains unclear.

**Methods:**

Single nucleotide polymorphisms (SNPs) associated with the serum levels of IGF-1 and the outcomes data of lung diseases including asthma, chronic obstructive pulmonary disease (COPD), lung cancer and idiopathic pulmonary fibrosis (IPF) were screened from the public genome-wide association studies (GWAS). Two-sample Mendelian randomization (MR) analysis was then performed to assess the independent impact of IGF-1 exposure on these lung diseases.

**Results:**

Totally, 416 SNPs related to circulating IGF-1 levels among 358,072 participants in UK Biobank. According to a primary casual effects model with MR analyses by the inverse variance weighted (IVW) method, the circulating IGF-1 was demonstrated a significantly related with the risk of asthma (OR, 0.992; 95% CI, 0.985-0.999, *P*=0.0324), while circulating IGF-1 showed no significant correlation with CODP (OR, 1.000; 95% CI, 0.999-1.001, *P*=0.758), lung cancer (OR, 0.979, 95% CI, 0.849-1.129, *P*=0.773), as well as IPIGFF (OR, 1.100, 95% CI, 0.794-1.525, *P*=0.568).

**Conclusion:**

The present study demonstrated that circulating IGF-1 may be causally related to lower risk of asthma.

## Introduction

Insulin-like growth factor 1 (IGF-1), a 70-amino acid polypeptide, is formerly known as somatomedin C for similarity of sequence and structure to insulin, which is mainly responsible for a series of biological functions, such as cell division, differentiation, apoptosis as well as metabolism (1, 2). The production of IGF-1 is mainly derived from the liver and controlled by growth hormone (GH), which also fulfils an endocrine function (3). IGF-1 acts primarily by binding to cysteine-rich regions of the IGF-I receptor (IGF-IR) subunit, which leads to conformational changes in the receptor that subsequently interact with adaptor proteins, such as members of the insulin receptor substrates (IRS), Granzyme B (GrB), and Src homology collagen (SHC) families, to initiate intracellular signal transduction cascades, containing phosphoinositol-3-kinas (PI3K)/protein kinase B (PKB/Akt) pathways and Ras-mitogen activated protein kinase, thereby involving in cell proliferation and apoptosis, respectively (4–6). Furthermore, IGF-1 can also influence metabolism of carbohydrate and lipid under the physiological and pathological conditions (7). Interestingly, metabolism of liver glucose may in turn directly regulate the transcription of IGF-1 gene (8).

Immunological and genetic analysis confirm the expression of IGF-1 in pathological or physiological lung tissue, such as airway cells, alveolar macrophages as well as lung fibroblasts. Lung fibroblasts have been shown to synthesize IGF-1 (9). Minuto et al. found that the expression of IGF-1 in tumor tissue were higher than in adjacent normal lung tissue (10). Moreover, activated alveolar macrophages can express IGF-1 (11). Taken together, IGF-1 may play a critical role in lung disease, especially in inflammatory disease, cancers, and lung fibrosis. However, so far, the relationship between circulating IGF-1 and lung diseases such as asthma, chronic obstructive pulmonary disease (COPD), lung cancer and idiopathic pulmonary fibrosis (IPF) remains unclear, and the available data cannot infer causality.

Mendelian randomization (MR) analysis, an epidemiological method, employs genetic variation as an instrumental variable for exposure, which can effectively reduce residual confusions and minimize reverse causal bias, thereby strengthening causal reasoning for exposion-outcome associations (12). At present, MR analysis has been favorably exerted to a wide range of observational associations, such as comprehending correlations between physiological indicators and evaluating the causal effects of various behaviors, especially the causal effects of biomarkers on diseases. In this context, we conducted MR analysis to determine the association between serum IGF-1 levels with asthma, COPD, lung cancers and IPF.

## Method and material

### Study design

**Figure 1:**
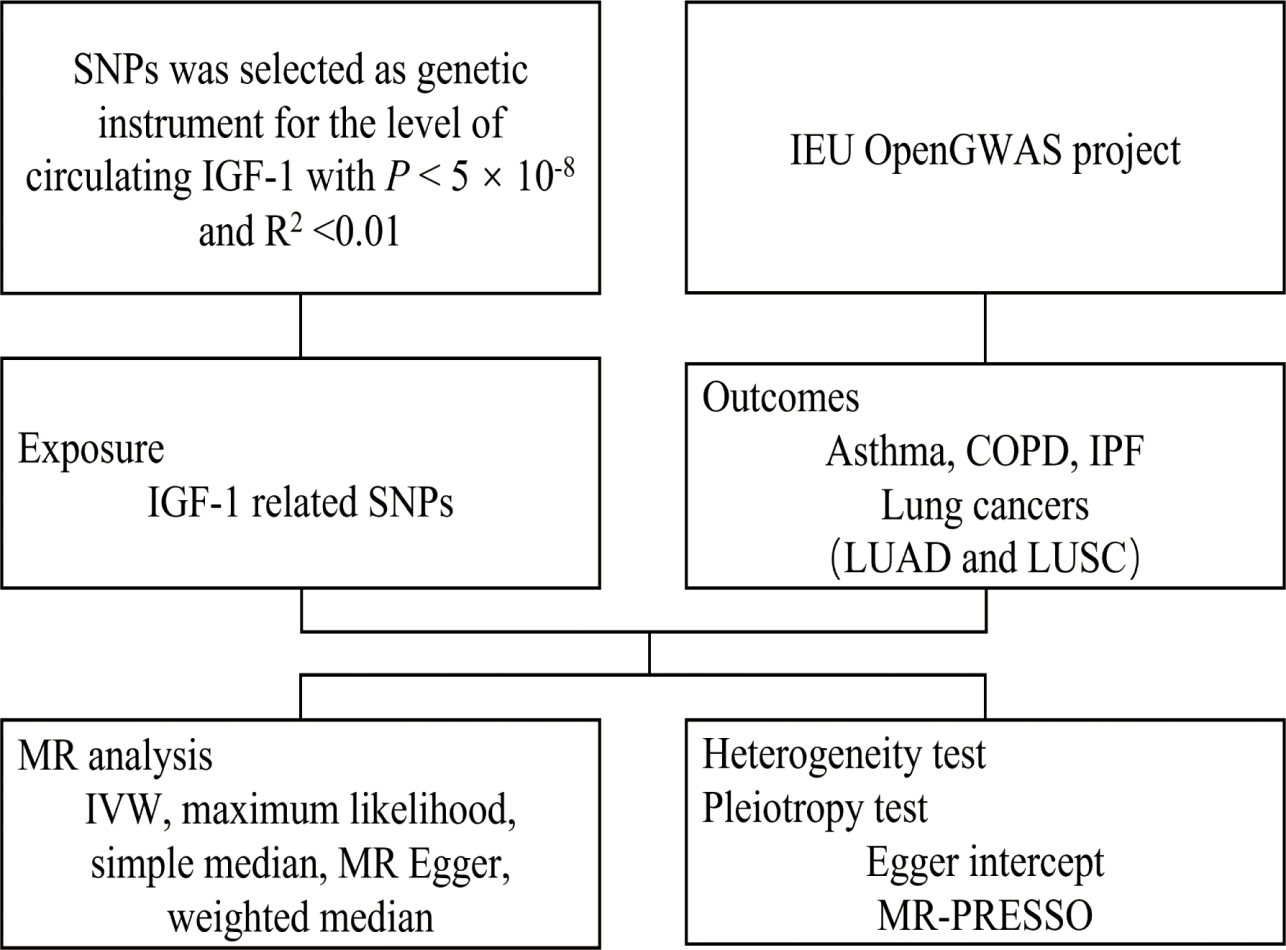
Flow chart of Mendelian randomization in this study.

The MR analysis uses genetic variation randomly assigned at meiosis and is therefore independent of potential confounders to act as a proxy for risk factors in the instrumental variable analysis. Beyond that, to be considered an effective tool, the gene variant must be closely related to the risk factor of interest and not directly influence the outcome, but only through exposure (13). Since observational studies tend to reverse causality and untested confounding, this study designed a two-sample MR analysis to investigate the causal effect of circulating IGF-1 levels on pulmonary diseases, including asthma, COPD, lung cancers, and IPF. Data on the correlation between single-nucleotide polymorphisms (SNPs) and circulating IGF-1 as well as the correlation between SNPs and pulmonary diseases were obtained from the GWAS database (**Figure 1**).

### Genetic instrument selection

Based on a GWAS containing 358072 participators of European descent from UK Biobank, a total of 416 SNPs was selected as genetic instrument for the level of circulating IGF-1 with *P* value of the genome-wide significant threshold less than 5 × 10^-8^ as well as a linkage disequilibrium threshold of R^2^ <0.01. The phenotypic variance in circulating IGF-1 levels explained by this genetic instrument was 9.4%, while the F-statistic of this genetic instrument reached 80.9 (14, 15).

### Data sources of lung diseases

The data associated with lung diseases in the present study were publicly available from Browse the IEU OpenGWAS project (mrcieu.ac.uk), which retrieves a series of large-scale GWAS. Summary-level data for asthma (GWAS ID: ukb-b-18113) were obtained from the MRC Integrative Epidemiology Unit at the University of Bristol (MRC-IEU) consortium with 53598 cases and 409335 non-cases. Summary-level data for COPD (GWAS ID: ukb-b-13447) were obtained from MRC-IEU consortium with 1605 cases and 461328 non-cases. Summary-level data for lung cancers (GWAS ID: ieu-a-966) were obtained from the international lung cancers consortium (ILCCO) with 11348 cases and 15681 non-cases. Furthermore, we subdivided lung cancer into lung adenocarcinoma (LUAD) and lung squamous cell carcinoma (LUSC), and analyzed the correlation between IGF-1 and them respectively. Summary-level data for IPF (GWAS ID: finn-b-IPF) were obtained with 1028 cases and 196986 non-cases. The above data are summarized in **Table 1**.

**Table 1:** The characteristics of GWAS data.

Disease	Samples size(case/control)	GWAS ID	Consortium	Years
Asthma	462933(53598/409335)	ukb-b-18113	MRC-IEU	2018
COPD	462933(1605/461328)	ukb-b-13447	MRC-IEU	2018
Lung cancers	27209(11348/15681)	ieu-a-966	ILCCO	2014
LUAD	18336(3442/14894)	ieu-a-965	ILCCO	2014
LUSC	18313(3275/15038)	ieu-a-967	ILCCO	2014
IPF	198014(1028/196986)	finn-b-IPF	NA	2021

### Statistical analysis

Inverse variance weighted (IVW) MR analyses based on the random effects model was used as the main analysis. IVW can be weighted to mean the approximate variance of the reciprocal of these single causal estimates (16). Cochran’s Q statistic is calculated to determine the heterogeneity between the analyses obtained from individual SNPs. Egger’s regression analysis and MR-PRESSO global test (17) were used to evaluate the potential directional pleiotropy. In addition to IVW, we also performed other methods to test the consistency of test results. Maximum likelihood is a traditional method with low standard error. It estimates the probability distribution function of the probability distribution parameter. Although there may be deviation sizes in a limited sample, the deviation is so small as to be biologically negligible (18). Simple median method, which offer consistent effect estimates if at least 50% of the genetic variation is a valid tool, while weight median method can account for differences in estimate accuracy and provide consistent estimates even if 50% of the information comes from invalid SNPs (19). All the results were considered statistically significant at *P* < 0.05 with two-tailed testing. All analyses were conducted in R (version 4.1.2) based on R package “TwoSampleMR”.

## Result

### IGF-1 and asthma

**Figure 2:**
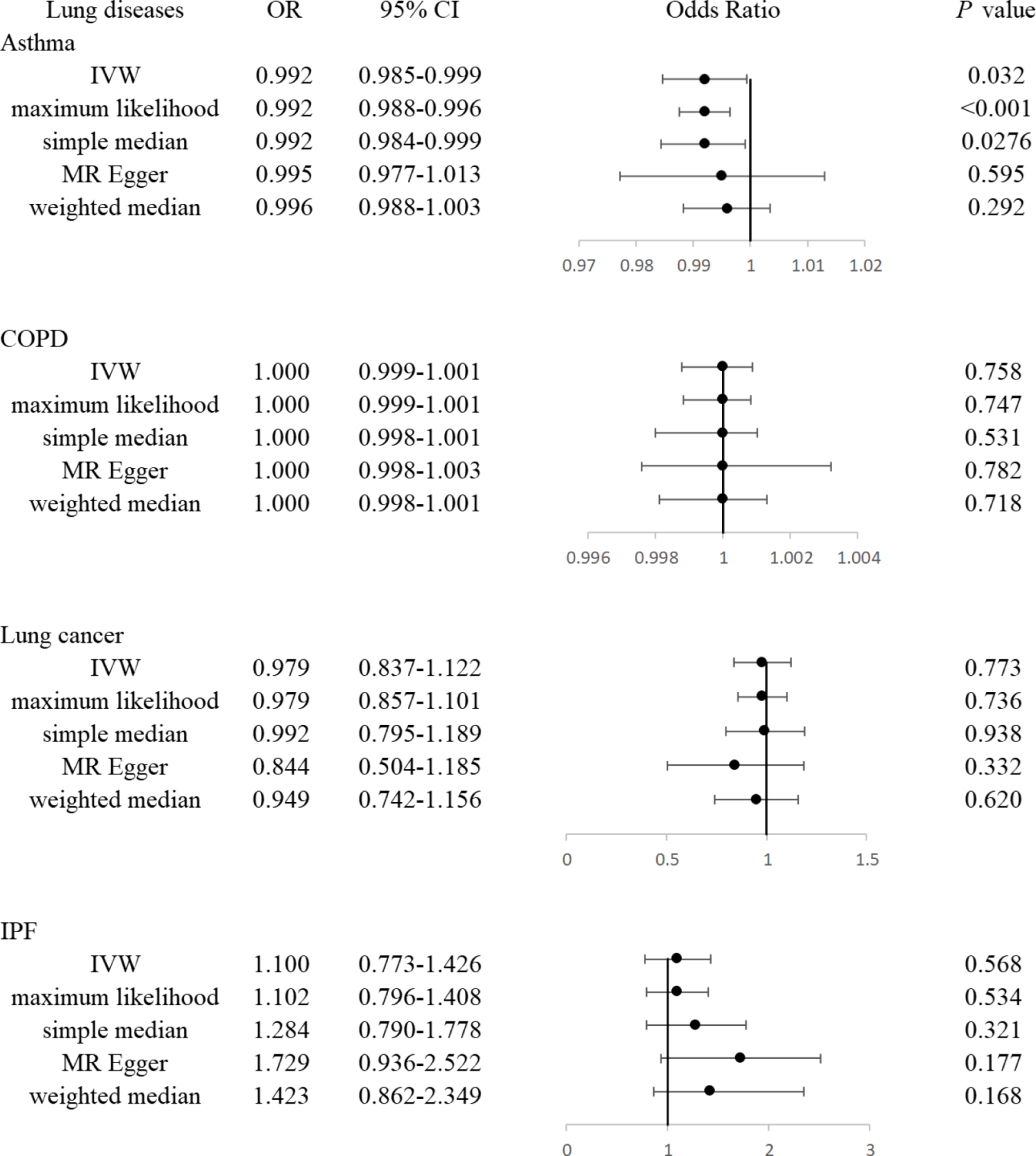
The forest plot showed associations of genetically predicted circulating IGF-1 with asthma, COPD, lung cancers, and IPF, respectively.

**Figure 3:**
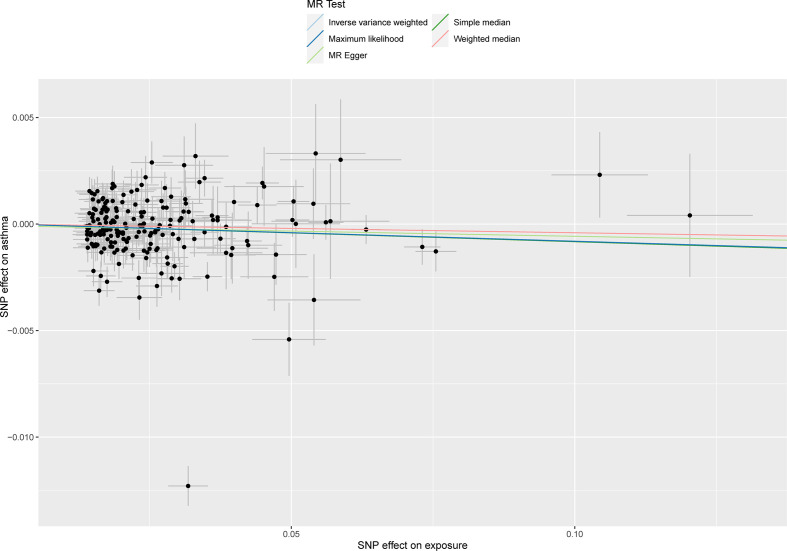
The scatter plot showed the genetic correlations between circulating IGF-1 and asthma using different MR methods. The slopes of line represent the causal effect of each method, respectively.

According to a primary casual effects model with MR analyses by the IVW method, IGF-1 has been demonstrated to be significantly associated with the risk of asthma (OR, 0.992; 95% CI, 0.985-0.999, *P*=0.0324, **Figure 2**). The results of maximum likelihood and simple median were consistent with IVW (OR, 0.992, 95%CI, 0.988-0.996, *P <*0.001; OR, 0.992, 95%CI, 0.984-0.999, *P* =0.0276, respectively). Although the results of MR Egger and weighted median showed that there was no statistically significant between IGF-1 exposure and asthma (OR, 0.995, 95%CI, 0.977-1.013, *P*=0.595; OR, 0.996, 95% CI, 0.988-1.004, *P*=0.292, respectively), the direction was in line with the results of the main analysis, especially IVW. Heterogeneity analysis suggested that there may be heterogeneity (*P*<0.001, **Table 2**), however, this did not affect the results of IVW and the conclusion was still reliable and acceptable. Moreover, horizontal pleiotropy was not found in the MR results (*P*=0.221). The scatter plot was presented in **Figure 3**, which showed the consistency of the results.

**Table 2:** The result of heterogeneity and pleiotropy test.

Disease	Heterogeneity *P* value	Pleiotropy *P* value	
		Egger intercept	MR-PRESSO
Asthma	<0.001	0.708	0.072
COPD	0.155	0.673	0.14
Lung cancer	<0.001	0.349	0.002
LUAD	0.012	0.258	0.014
LUSC	<0.001	0.416	<0.001
IPF	0.0732	0.221	0.083

### IGF-1 and COPD

The IVW method indicated that IGF-1 showed no significant correlation with CODP (OR, 1.000; 95% CI, 0.999-1.001, *P*=0.758). Similar to the IVW method, the result of maximum likelihood, simple median, MR Egger and weighted median showed the consistent consequence (OR, 1.000, 95% CI, 0.999-1.001, *P*=0.747; OR, 1.000, 95% CI, 0.998-1.001, *P*=0.531; OR, 1.000, 95% CI, 0.998-1.003, *P*=0.782; OR, 1.000, 95% CI, 0.998-1.001, *P*=0.718, respectively). The heterogeneity and horizontal pleiotropy have not been found in these analyses.

### IGF-1 and lung cancers

The IVW method show no association between the IGF-1 and lung cancers (OR, 0.979, 95% CI, 0.849-1.129, *P*=0.773), as well as maximum likelihood, simple median, MR Egger and weighted median showed the consistent consequence (OR, 0.979, 95% CI, 0.857-1.101, *P*=0.736; OR, 0.992, 95% CI, 0.795-1.189, *P*=0.938; OR, 0.844, 95% CI, 0.601-1.187, *P*=0.332; OR, 0.949, 95% CI, 0.771-1.167, *P*=0.620, respectively). The analysis between IGF-1 and LUAD as well as LUSC showed the similar results (**Table S1**). Notably, although heterogeneity tests suggested the existence of heterogeneity, the results were still reliable. There was no evidence of directional pleiotropy existing according to MR-Egger intercept, while MR-PRESSO global test showed the contrary result. We further discovered that the outlier-corrected analysis showed similar results (**Table S2**).

### IGF-1 and IPF

All the MR results showed that the IGF-1 had no significant genetic correlation with IPF (OR, 1.100, 95% CI, 0.794-1.525, *P*=0.568 for IVW; OR, 1.102, 95% CI, 0.912-1.292, *P*=0.535 for maximum likelihood; OR, 1.284, 95% CI, 0.794-1.774, *P*=0.321 for simple median; OR, 1.729, 95% CI, 0.783-3.821, *P*=0.177 for MR Egger; OR, 1.423, 95% CI, 0.862-2.349, *P*=0.168 for weighted median, respectively). The heterogeneity and horizontal pleiotropy have not been found in these analyses.

## Discussion

In the present study, the two-sample MR analysis applying a series of large sample GWAS data indicated that the level of circulating IGF-1 may contribute to the inverse risk of asthma, while there was no obvious evidence to testify the relationship between the level of IGF-1 and COPD, lung cancers as well as IPF. Interestingly, despite the lack of epidemiological as well as clinical studies of circulating IGF-1 in relation to lung disease, substantial preclinical evidence has revealed the potential effects of IGF-1 in the pathobiology of asthma, COPD, lung cancers, and IPF.

Our findings showed consistency with several but not all previous studies of the role of IGF-1 in asthma. A study among British population cohort suggested that the level of serum IGF-1 was associated with a lower risk of asthma, which was in line with our study (20). However, a case-control study of 50 participants reported a positive association of serum IGF-1 with asthma. Nevertheless, the study was limited by a small sample size (21). Indeed, IGF-1 may play a crucial role in asthma, especially in airway hyperresponsiveness, airway inflammation, as well as smooth airway hyperplasia. A study of bronchial biopsy in asthmatic patients showed significantly elevated mRNA levels of IGF-1 and was associated with subepithelial fibrosis (22). These studies suggested that IGF-1 may involve in airway inflammation and remodeling. Supporting this hypothesis is the expression of airway IGF-1 was upregulated in ovalbumin (OVA)-induced asthmatic mouse models, while administration of anti-IGF-1 improved airway the inflammation, resistance, and wall thickening of airway (23). Upregulated IGF-1 in the lungs of asthmatic mice was confirmed to mainly originate from alveolar macrophages (24). Furthermore, IGF-1 can suppress alveolar epithelial cells from phagocytosis of apoptotic cells, thereby promoting the release of inflammatory contents of apoptotic cells and resulting in the increased airway inflammation (25). Thus, observations from preclinical experimental studies implied that increased IGF-1 signaling may aggravate asthma risk, while findings from this study as well as other epidemiological studies indicated that IGF-1 may be associated with reduced asthma risk. The apparently contradictory results regarding IGF-1 and asthma can be explained in part by the fact that preclinical models measured IGF-1 in the lungs, while epidemiological studies assessed circulating serum IGF-1 levels, which may have disparate impacts on and airway inflammation.

In a retrospective study of 61 patients with COPD, the level of serum IGF-1 were significantly lower in COPD patients compared with that in controls. Moreover, circulating IGF-1 levels were also significantly lower in patients with acute exacerbation of COPD (AECOPD) than that in patients with clinically stable COPD (AECOPD) (26). These results were inconsistent with our research, which may be partly due to sample size.

For the relationship between IGF-1 and IPF, there is currently little research, especially population-based epidemiological investigations. To our knowledge, the present study first demonstrated that the level of serum IGF-1 was irrelevant with IPF from a population-based perspective. Early report has suggested that the IGF-1 in bronchoalveolar lavage (BAL) fluids was involved in IPF (27). Mechanically, TGF-β may enhance the activation of IGF-1 to promote pulmonary fibrosis (28). In contrast to this result, Bloor et al. found that the total expression of IGF-1 was downregulated in BAL cells in patients with IPF compare to the healthy subjects (29). It was hypothesized that TGF-β enhancement not only leaded to the synthesis of fibroblast extracellular matrix and differentiation of myofibroblasts, but also enhanced epithelial cell death by reducing the expression of IGF-1 and the secretion of apoptotic factors. As a result, fibroblast repair time may be prolonged and epithelial cells were unable to regenerate, thus promoting fibrotic scarring and further inducing pulmonary fibrosis (6). In addition, temporal regulation and spatial localization of IGF-1 production may play an important role in the progression of lung disease (30).

In an analysis of IGF-1 and pan-cancer, circulating IGF-1 was not associated with lung cancers, which matched our results (31). However, the IGF-1 signaling was involved in virtually all stages of lung cancers. For example, severe bronchial dysplasia produces more paracrine and autocrine IGF-1 than benign bronchial epithelial cells, which then interacts with tobacco carcinogens to promote lung carcinogenesis, thereby implying an early role of IGF-1 in the development of lung cancers (32). It was also found that exogenous IGF-1 induced the upregulation of epithelial-mesenchymal transition (EMT) and promoted the proliferation, invasiveness, metastasis, and eventually resistance to epidermal growth factor receptor-tyrosine kinase inhibitors (EGFR-TKIs) *via* binding IGF-1 receptor (33). The apparent contradiction between IGF-1 and lung disease in preclinical studies and epidemiology can be partly explained by the fact that preclinical models primarily measure IGF-1 in the lungs, as mentioned in asthma and IPF, while epidemiological studies primarily assess the level of peripheral serum IGF-1, which may have different effects on immune response and airway inflammation. Furthermore, feedback between IGF-1 and T helper cells-associated cytokines may lead to conflicting results from experimental and epidemiological studies. It has been reported that IL-4 and IL-13 can enhance the expression of IGF-1 induced by IL-17 *in vitro* (34). On the other hand, negative feedback between proinflammatory cytokines and IGF-1 has been suggested. Tumor necrosis factor (TNF)-α and IL-1β can decrease the sensitivity of IGF-1 by enhancing IGFBP production and impeding IGF-1 binding to its receptor IGF-1R through an insulin receptor substrate (IRS)-Akt pathway. Relatively, IGF-1 can suppress proinflammatory cytokine signaling by c-Jun N terminal kinase (JNK) and NF-κB pathways as well as increasing the secretion of IL-10 (35). Notably, smoking may influence the level of IGF-1, as smokers squint towards having lower circulating IGF-1 levels than non-smokers (36).

The current study has several strengths and limitations. The main advantage is that the MR analysis can reduce the bias from residual confounding and reverse causality, thus strengthening the comprehensive assessment between circulating IGF-1 and pulmonary diseases. In addition, the genetic instrument of IGF-1 has favourable validity and has been used in previous MR studies, which guarantees the robustness of our results. Several limitations in this MR study should be warrant mentioned. First, the information on the grade of asthma, COPD, and lung cancers was not available, so we cannot examine whether the association with IGF-1 was different based on these characteristics. Second, more than 400 SNPs were used as genetic instrument of IGF-1 in this study, which may increase the possibility of bias caused by invalid instruments. Nevertheless, supporting results from other sensitivity analyses reduce this possibility and validate our findings. Third, epigenetic phenomena, such as methylation, and interactions between genes and environmental exposure may also influence IGF-1 in relation to lung diseases. However, we were unable to assess these effects in the current MR study.

## Conclusion

Together, these MR Results support the possibility of a causal relationship between elevated serum IGF-1 levels and asthma, and these conclusions are useful for clinical detection in patients with asthma.

## Data availability statement

The datasets presented in this study can be found in online repositories. The names of the repository/repositories and accession number(s) can be found below: https://gwas.mrcieu.ac.uk.

## Author contributions

KW and LD designed the study. SJ and YS collected and analyzed the data. SL, MX and XJ repared the tables and figures. SJ and ZX wrote the paper. All authors reviewed and approved the manuscript.

## Supplementary material

Supplementary Table 1 The MR analysis of LUAD and LUSC.

Supplementary Table 2 MR-PRESSO outlier-corrected Analysis for Lung cancers.
