# Supplementary material for: Circulating insulin-like growth factor-1 and risk of lung diseases: A Mendelian randomization analysis
Source: Front Endocrinol (Lausanne). 2023 Mar 3;14:1126397. doi: 10.3389/fendo.2023.1126397 (PMC10020499; doi:10.3389/fendo.2023.1126397)
Supplement: Supplementary Table 1 — The MR analysis of LUAD and LUSC. [file Table_1.docx]

Supplementary Table S1 The MR analysis of LUAD and LUSC

| Lung diseases | OR | 95% CI | *P* value |
| --- | --- | --- | --- |
| LUAD |  |  |  |
| IVW | 0.899 | 0.744-1.104 | 0.309 |
| maximum likelihood | 0.898 | 0.777-1.083 | 0.254 |
| simple median | 0.919 | 0.617-1.201 | 0.557 |
| MR Egger | 0.698 | 0.263-1.181 | 0.146 |
| weighted median | 0.897 | 0.667-1.202 | 0.483 |
| LUSC |  |  |  |
| IVW | 0.965 | 0.774-1.119 | 0.752 |
| maximum likelihood | 0.964 | 0.777-1.151 | 0.704 |
| simple median | 0.921 | 0.617-1.224 | 0.595 |
| MR Egger | 0.791 | 0.263-1.318 | 0.384 |
| weighted median | 0.985 | 0.667-1.303 | 0.927 |
