# Supplementary material for: Circulating insulin-like growth factor-1 and risk of lung diseases: A Mendelian randomization analysis
Source: Front Endocrinol (Lausanne). 2023 Mar 3;14:1126397. doi: 10.3389/fendo.2023.1126397 (PMC10020499; doi:10.3389/fendo.2023.1126397)
Supplement: Supplementary Table 2 — MR-PRESSO outlier-corrected Analysis for Lung cancers. [file Table_2.docx]

Supplementary Table S2 MR-PRESSO outlier-corrected Analysis for Lung cancers

| Outcomes | Exposure | MR-PRESSO | *p*-value |
| --- | --- | --- | --- |
| Lung cancers | IGF-1 | Outlier-corrected |  |
| LUAD |  |  | 0.640 |
| LUSC |  |  | 0.664 |
